# Overexpression of IL-1ra gene up-regulates interleukin-1**β** converting enzyme (ICE) gene expression: possible mechanism underlying IL-1**β**-resistance of cancer cells

**DOI:** 10.1038/sj.bjc.6690688

**Published:** 1999-09

**Authors:** M Sumitomo, M Tachibana, M Murai, M Hayakawa, H Nakamura, A Takayanagi, N Shimizu

**Affiliations:** Department of Urology, School of Medicine,Keio University, Tokyo, Japan; Department of Urology, National Defense Medical College, Tokorozawa, Saitama, Japan; Department of Molecular Biology, School of Medicine, Keio University, Tokyo, Japan

**Keywords:** IL-1β, IL-1ra, ICE, adenovirus vector, apoptosis

## Abstract

We investigated the interaction of endogenous interleukin (IL)-1β, IL-1ra, and interleukin-1β converting enzyme (ICE) in four human urological cancer cell lines, KU-19-19, KU-1, KU-2 and KU-19-20. Northern blot analysis showed that IL-1β gene was expressed in all cell lines. On the other hand, in KU-19-19 and KU-19-20, the gene expressions of both IL-1ra and ICE were suppressed. MTT assay revealed that IL-1β (10 ng ml^−1^) promoted cell growth in KU-19-19 and KU-19-20, while it inhibited in KU-1 and KU-2. An ICE inhibitor, Acetyl-Tyr-Val-Ala-Asp-CHO (YVAD-CHO) blocked IL-1β-induced growth inhibition in KU-1 and KU-2. Overexpression of the secretory type IL-1ra with adenovirus vector (AxIL-1ra) enhanced ICE gene expression, while exogenous IL-1ra (100 ng ml^–1^) did not enhance it. Furthermore, AxIL-1ra treatment promoted endogenous IL-1β secretion and induced significant growth inhibition and apoptotic cell death on KU-19-19 and KU-19-20. Treatment with either IL-1ra (100 ng ml^−1^), IL-1β antibody (100 μg ml^−1^), or YVAD-CHO blocked AxIL-1ra-induced cell death in KU-19-19 and KU-19-20. These results suggest that IL-1β-sensitivity depends on the level of ICE gene expression, which is regulated by the level of endogenous sIL-1ra expression. This is a first report on the intracellular function of sIL-1ra and these findings may provide key insights into the mechanism underlying the viability of cancer cells. © 1999 Cancer Research Campaign


*British Journal of Cancer* (1999) **81**(2), 277–286
© 1999 Cancer Research Campaign
Article no. bjoc.1999.0688

### Overexpression of IL-1ra gene up-regulates interleukin-1β converting enzyme (ICE) gene expression: possible mechanism underlying IL-1β-resistance of cancer cells

**M Sumitomo[1,2], M Tachibana[1], M Murai[1], M Hayakawa[2], H Nakamura[2], A Takayanagi[3] and N Shimizu[3]**

[1]Department of Urology, School of Medicine, Keio University, Tokyo, Japan; [2]Department of Urology, National Defense Medical College, Tokorozawa, Saitama, Japan; [3]Department of Molecular Biology, School of Medicine, Keio University, Tokyo, Japan

**Summary** We investigated the interaction of endogenous interleukin (IL)-1β, IL-1ra, and interleukin-1β converting enzyme (ICE) in four human urological cancer cell lines, KU-19-19, KU-1, KU-2 and KU-19-20. Northern blot analysis showed that IL-1β gene was expressed in all cell lines. On the other hand, in KU-19-19 and KU-19-20, the gene expressions of both IL-1ra and ICE were suppressed. MTT assay revealed that IL-1β (10 ng ml$^{-1}$) promoted cell growth in KU-19-19 and KU-19-20, while it inhibited in KU-1 and KU-2. An ICE inhibitor, Acetyl-Tyr-Val-Ala-Asp-CHO (YVAD-CHO) blocked IL-1β-induced growth inhibition in KU-1 and KU-2. Overexpression of the secretory type IL-1ra with adenovirus vector (AxIL-1ra) enhanced ICE gene expression, while exogenous IL-1ra (100 ng ml$^{-1}$) did not enhance it. Furthermore, AxIL-1ra treatment promoted endogenous IL-1β secretion and induced significant growth inhibition and apoptotic cell death on KU-19-19 and KU-19-20. Treatment with either IL-1ra (100 ng ml$^{-1}$), IL-1β antibody (100 μg ml$^{-1}$), or YVAD-CHO blocked AxIL-1ra-induced cell death in KU-19-19 and KU-19-20. These results suggest that IL-1β-sensitivity depends on the level of ICE gene expression, which is regulated by the level of endogenous sIL-1ra expression. This is a first report on the intracellular function of sIL-1ra and these findings may provide key insights into the mechanism underlying the viability of cancer cells.

© 1999 Cancer Research Campaign

**Keywords:** IL-1β; IL-1ra; ICE; adenovirus vector; apoptosis

The interleukin-1 (IL-1) family of cytokines consists of structurally and functionally related molecules with pleiotropic activities involved in immune and inflammatory responses (Dinarello, 1996). IL-1β, one member of this family, has been implicated in a wide range of physiological and pathological processes, including mitogenic T-cell stimulation, wound healing, cellular adhesion, cytokine production, inflammation and sepsis (Oppenheim et al, 1986; Dinarello and Thompson, 1991; Dinarello and Wolff, 1993). Other members of this family include IL-1α and a naturally occurring antagonist, referred to as IL-1 receptor antagonist (IL-1ra), which share sequence homologies and the usage of similar receptors (Dinarello and Thompson, 1991). IL-1β is intracellularly cleaved and exported in large quantities after the stimulation of its producer cell, whereas IL-1α is synthesized in much lower quantities and does not appear to be actively secreted (Hazuda et al, 1988; Grassi et al, 1991). IL-1ra is now known to exist in two forms, a secretory product (sIL-1ra) and an intracellular molecule (icIL-1ra) found in the cytoplasm (Dinarello, 1996). Both forms of IL-1ra are encoded from the same gene but their transcription is regulated by different promoter regions.

IL-1β is synthesized as an inactive 33 kDa precursor protein (pIL-1β) that is cleaved by a protease to generate the mature 17-kDa secretory protein (mIL-1β) (Kostura et al, 1989; Lonnenmann et al, 1989). A cystein protease responsible for the cleavage of IL-1β has been identified and termed IL-1β converting enzyme (ICE) (Black et al, 1989; Cerretti et al, 1992). Several enzymes sharing sequence homology and the ability to cleave proteins at an asparagine-site have been identified over the past few years and were recently named caspases (Alnemri et al, 1996). Besides ICE, which was assigned to the name caspase-1, nine other caspases are known, several of which are known to be involved in the regulation of apoptosis. However, it has recently been shown that ICE does not play a requisite role in Fas-mediated apoptosis, and thus the role of ICE during apoptosis remains unclarified (Enari et al, 1996; Tatsuda et al, 1996; Smith et al, 1997).

IL-1 regulates the proliferation of many cell types both in a positive and in a negative manner. IL-1 stimulates the growth of thymocytes, B-cells, fibroblasts and a human glioma cell line (Gery et al, 1972; Schmidt et al, 1982; Lachman et al, 1987; Freedman et al, 1988). In contrast, IL-1 is antiproliferative for a human melanoma cell line (Onozaki et al, 1985), several malignant human mammary cell lines (Gaffney and Tsai, 1986) and human endothelial cells (Maier et al, 1990). Surprisingly, several authors have reported that endogenous IL-1β regulates both cell proliferation and cell death in some cell lines (Fratelli et al, 1995; Friedlander et al, 1996). The mechanism underlying the sensitivity to IL-1 has only been partially clarified. Araki et al (1994) reported that resistance to the antiproliferative effect of IL-1 is associated with endogenous IL-1 production. Friedlander et al (1996) reported that the ICE down-regulates IL-1β-IL-1 receptor-binding activity, which causes the resistance to IL-1β. Furthermore, many authors have recently reported that endogenous IL-1 and IL-1ra balance regulates cell proliferation in many cell types (Corradi et al, 1995; Furukawa et

*Received 14 January 1999*
*Revised 7 April 1999*
*Accepted 9 April 1999*

*Correspondence to:* M Tachibana, Department of Urology, School of Medicine, Keio University, 35 Shinanomachi, Shinjuku-ku, Tokyo 160-8582, Japan al, 1995; Oelmann et al, 1997), although it remains unclear how this balance can be regulated in these cells. Thus, both IL-1ra and ICE seem to be closely associated with IL-1-mediated cell proliferation and/or cell death.

We have previously reported that the bladder cancer cell line KU-19-19 produces multiple cytokines; IL-6, IL-8, granulocyte-colony stimulating factor (G-CSF) and granulocyte–macrophage colony stimulating factor (GM-CSF) (Tachibana et al, 1997*a*). IL-1 is well known as one of the key cytokines that can promote the production of these cytokines (Dinarello, 1996). In cancer cells such as KU-19-19, that produce multiple cytokines regulated by IL-1, it is thought that IL-1 plays a crucial role in both the production of various cytokines and cell proliferation.

In the present study, we hypothesized that endogenous IL-1β and IL-1ra balance could regulate the ICE expression. We demonstrated that the gene expressions of both ICE and IL-1ra were suppressed and IL-1β secretion was inhibited in IL-1β-resistant cancer cell lines. Furthermore, we demonstrated that the over-expression of sIL-1ra could enhance ICE gene expression, which induced the cleavage and secretion of intracellular pIL-1β and promoted IL-1β-mediated cell death, while exogenous IL-1ra could not enhance it. These findings may provide the key clues to clarify the interaction of endogenous IL-1β, IL-1ra and ICE.

### MATERIALS AND METHODS

### Reagents

Recombinant human IL-1β (specific activity; $1 \times 10^9$ units per mg) was purchased from Genzyme (Boston, MA, USA) and recombinant human IL-1ra (specific activity; $5 \times 10^5$ units per mg) was purchased from Anapure Bioscientific Co. Ltd (Beijing, China). Rabbit anti-human IL-1β monoclonal antibody and rabbit anti-human IL-1ra antibody were purchased from Genzyme. Acetyl-Tyr-Val-Ala-Asp-CHO (YVAD-CHO) was obtained from Sigma (St Louis, MO, USA).

### Recombinant adenovirus vectors

We constructed a recombinant adenovirus vector expressing sIL-1ra (Adex1CAKTIL-1ra; abbr.: AxIL-1ra). A modification of method (COS-TPC method) developed by Dr Saito et al (Miyake et al, 1996), was used to construct this adenovirus vector. In brief, this replication-deficient adenovirus is based on adenovirus type 5 which lacks the E1A, E1B and E3 regions of the virus and contains the CAG promoter, sIL-1ra cDNA, and poly-adenosine (poly-A) signal sequences inserted into the E1-deleted region. The sIL-1ra cDNA, amplified by RT-PCR using the total RNA obtained from U937 cells stimulated by 10 ng $ml^{-1}$ TPA for 20 h and the oligo-dT primer and the following primers, 5′- GCCACCATGGAAATCTGCAGAG-GCCTCCGCAGTC-3′ and 5′-GCTCTAGACTACTCGTCCTC-CTGGAAGTAGAATTTGG-3′, was end-blunted with T4 DNA polymerase, digested with *Xba*I, cloned into *Sca* I-*Xba* I site of pBK-CMV, and sequenced. The *Hin*d III-*Sma* I fragment of the cDNA was inserted into the unique *Swa* I site of adenovirus genome in the cassette cosmid, pAdex1CA1wt. The cosmid bearing the expression unit in adenovirus genome was then co-transfected into human embryonic kidney 293 cells together with the adenovirus DNA–terminal protein complex (DNA-TPC) digested at seven sites with *Eco*T22 I. Purified virus stocks were prepared by CsCl step gradient centrifugation as previously described method (Kanegae et al, 1994). As a control vector, recombinant lacZ adenovirus (Adex1CALacZ; abbr.: AxLacZ), containing the CAG promoter, lacZ gene, and poly-A signal sequences, was kindly supplied by Dr Saito et al (Kanegae et al, 1995). Almost all of the cancer cells infected with AxLacZ at 5 MOI demonstrated lacZ expression (data not shown).

### Cell culture

Four different urological cancer cell lines derived from human bladder cancer cells: KU-19-19 and KU-1 (Tachibana, 1982; Tachibana et al, 1997*b*), and human renal cancer cells: KU-2 (Katsuoka et al, 1976) and KU-19-20 were used. KU-19-19 (Tachibana et al, 1995, 1997*a*, 1997*b*) has recently been established from the advanced bladder cancer patient and KU-19-20 (unpublished data) has also recently been established from the advanced renal cell carcinoma patient. These two patients had marked leucocytosis. We have found that both KU-19-19 and KU-19-20 produce multiple cytokines, IL-6, IL-8, G-CSF and GM-CSF. Furthermore, G-CSF induces autocrine growth on KU-19-19 (Tachibana et al, 1995) and GM-CSF induces autocrine growth on KU-19-20 (unpublished data), while neither KU-1 nor KU-2 produces multiple cytokines. These cancer cell lines were routinely propagated in a monolayer culture in a humidified incubator at 37°C in a 5% carbon dioxide/95% air atmosphere. Cells were grown in RPMI-1640 medium supplemented with 10% heat-inactivated fetal bovine serum (FBS), 100 μg $ml^{-1}$ streptomycin (Gibco-BRL, Grand Island, NY, USA) and 100 IU $ml^{-1}$ penicillin (Gibco-BRL).

### Western blot analysis

Cells ($1 \times 10^6$) were homogenized in 100 μl of lysis buffer (20 mM Tris–HCl pH 8.0, 137 mM sodium chloride, 10% glycerol, 1% NP-40, 1 mM phenylmethylsulphonyl fluoride (PMSF; Sigma)), incubated at 4°C for 20 min, and then were centrifuged at 10 000 ***g*** for 15 min at 4°C to remove debris. The protein concentrations in the cell lysates were determined by the use of bicinconinic acid (BCA) protein assay reagent (Pierce, Rockford, IL, USA), and then the lysates were mixed with sample buffer containing 2-mercaptoethanol and boiled for 5 min. Samples containing 30 μg total protein were subjected to sodium dodecyl sulphate polyacrylamide gel electrophoresis (SDS-PAGE) using a 14% polyacrylamide gel (Multigel, Daiichi Pure Chemicals, Tokyo, Japan), and then were transferred onto a polyvinylidene difluoride (PVDF) membrane (Immobilon, Daiichi Pure Chemicals, Tokyo, Japan) by semi-dry electroblotting. The membrane was blocked for 2 h at 37°C in phosphate-buffered saline (PBS) with 0.05% Tween-20 (PBT), containing 1% bovine serum albumin (BSA). Either rabbit anti-IL-1β monoclonal antibody or anti-IL-1ra antibody was added at a concentration of 10 μg $ml^{-1}$ and incubated for 2 h at 37°C. Immunoreactive polypeptides were detected by using donkey anti-rabbit immunoglobulin G conjugated to horseradish peroxidase in an enhanced chemiluminescence system according to the manufacturer's protocol (Amersham Pharmacia Biotech, Buckinghamshire, UK).

### Northern blot analysis

The total RNA from each cancer cell was isolated by acid–guanidinium–thiocyanate–phenol–choloroform extraction. The concen-

© 1999 Cancer Research Campaign

**Table 1** The concentrations of several cytokines in the supernatants of cancer cells

| | IL-1β | IL-1ra | IL-1ra/IL-1β ratio | IL-6 | IL-8 | G-CSF | GM-CSF |
|---|---|---|---|---|---|---|---|
| KU-19-19 | 27.2 ± 5.1 | 84.5 ± 10.1 | 3.1 | 64.9 ± 14.1 | 487.5 ± 32.8 | 519.7 ± 58.3 | 191.8 ± 29.0 |
| KU-1 | 52.4 ± 9.4 | 726.7 ± 64.1 | 13.9 | 0.5 ± 0.1 | ND | ND | ND |
| KU-2 | 79.3 ± 8.6 | 854.9 ± 43.6 | 10.7 | 0.7 ± 0.1 | ND | ND | ND |
| KU-19-20 | 15.6 ± 4.6 | 74.6 ± 11.5 | 4.8 | 91.3 ± 12.4 | 395.7 ± 44.7 | 181.0 ± 21.4 | 398.3 ± 61.1 |

(pg $ml^{-1}$)

Cells ($1 \times 10^4$ per well) were incubated in a 1-ml culture medium for 48 h and the concentrations of several cytokines in the supernatants were measured by ELISA. The concentrations of both IL-1β and sIL-1ra in the supernatants and IL-1ra/IL-1β ratios were higher in KU-1 and KU-2 than in KU-19-19 and KU-19-20. On the other hand, IL-6, IL-8, G-CSF and GM-CSF were secreted in large quantities in both KU-19-19 and KU-19-20, but not in either KU-1 or KU-2. The results are expressed as the mean ± s.d. of triplicate samples. ND, not detectable. The experiments were repeated three times with similar results.

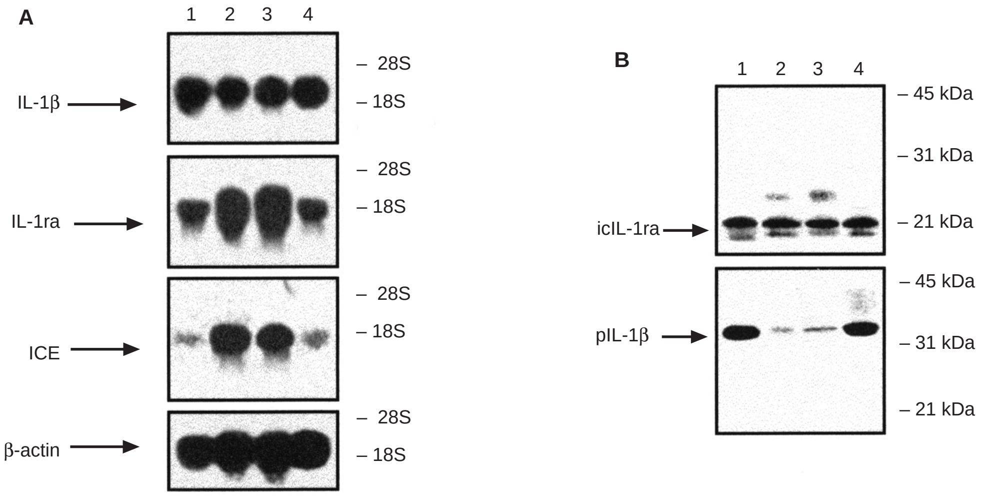

**Figure 1** The levels of gene expression and production of IL-1β, IL-1ra and ICE in four cancer cells. (**A**) Detection of gene expression by Northern blot analysis. Cells were incubated for 48 h and total RNA samples (20 μg) were size-fractioned by electrophoresis in 1% formaldehyde agarose gel. The RNA was transferred and hybridized by the 30-mer antisense oligonucleotide probes for IL-1β, IL-1ra and ICE as described in Materials and Methods. A 27-mer antisense oligonucleotide probe for human β-actin was used as a loading control. IL-1β gene was expressed at the same levels in all cancer cells. In contrast, the levels of IL-1ra and ICE gene expression were suppressed more in KU-19-19 (lane 1) and KU-19-20 (lane 4) than in KU-1 (lane 2) and KU-2 (lane 3). (**B**) Detection of intracellular IL-1β and IL-1ra by Western blot analysis. Cells were incubated for 48 h and cells ($1 \times 10^6$) were lysed and 30 μg of each lysate was subjected to SDS-PAGE, transferred onto a polyvinylidene difluoride membrane, and immunoblotted with rabbit anti-IL-1β antibody or anti-IL-1ra antibody and donkey anti-rabbit immunoglobulin G conjugated to horseradish peroxidase as described in Materials and Methods. The intracellular form of IL-1ra (icIL-1ra, about 20 kDa) was detected in all cancer cells. In KU-19-19 (lane 1) and KU-19-20 (lane 4), the bands corresponding to immature form pIL-1β (about 33 kDa) were strongly detected, while smaller bands were detected in KU-1 (lane 2) and KU-2 (lane 3). These blots are representative samples of two experiments showing similar tendencies tration of total RNA was assessed by optimal density readings at 260 nm. The total RNA samples (20 μg) were size-fractioned by electrophoresis in 1% formaldehyde agarose gel. The RNA was transferred overnight to nylon filters (Hybond-N; Amersham) by capillary diffusion in 20 × SSC (1 × SSC is 0.15 mol sodium chloride (NaCl) and 0.015 mol sodium citrate $1^{-1}$) and then baked at 80°C in a vacuum for 2 h. The 30-mer antisense oligonucleotide probes for IL-1β (nucleotide numbers 787–816), IL-1ra (nucleotide numbers 342–371) and ICE (nucleotide numbers 664–693) were 3’-end labelled with [$\alpha^{32}$P] dCTP (6000 Ci $mmol^{-1}$; Amersham) using terminal transferase (Terminal Transferase 500 U; Boehringer Mannheim, Germany) and purified through a Sephadex G50 NIC™ Column (Pharmacia Biotech, Uppsala, Sweden). The filters were prehybridized at 65°C for 1–2 h in hybridization buffer containing 10% dextran sulphate, 1 M NaCl, 25 μg yeast torula RNA $ml^{-1}$ (Boehringer Mannheim) and 1% SDS, and then were hybridized overnight at 65°C with $2 \times 10^6$ ct $min^{-1}$ labelled probes $ml^{-1}$. A 27-mer antisense oligonucleotide probe for human β-actin was used as a loading control (Toyobo, Tokyo, Japan). The hybridized filters were washed three times for 20 min at 60°C in 0.1 × SSC with 0.1% SDS. Thereafter, the filters were subjected to autoradiography.

### Cell growth assay

Cells were incubated in a flat-bottom 96-well plate (Falcon; Becton Dickinson Labware, Lincoln Park, NJ, USA) for different time periods. At the end of the experiment, 20 μl of the dye MTT (3,(4,5-dimethylthiazol-2-y1-) diphenyltetrazolium bromide 5 mg $ml^{-1}$) was added to each well and the plates were incubated for 3 h at 37°C. Then, 100 μl of lysis buffer (20% SDS in 50% *N*,*N*-dimethylformamide, containing 0.5% [v:v] 80% acetic acid and 0.4% [v:v] 1 N hydrochoric acid were added to each well and the colour intensity (proportional to the number of live cells) was assessed using a microplate reader at 570 nm wavelength. Each experiment was performed in triplicate.

*© 1999 Cancer Research Campaign* 

### Determination of the concentrations of IL-1β and several cytokines by ELISA

Cells ($1 \times 10^4$ per well) were incubated in a 1 ml culture medium for different time periods. Following incubation, each cell culture was centrifuged and the concentrations of IL-1β, IL-1ra, IL-6, IL-8, G-CSF and GM-CSF in the supernatants were measured by enzyme-linked immunosorbent assay (ELISA). Each experiment was performed in triplicate.

### Fragmented DNA detection by ELISA

The presence of fragmented DNA was assayed by specific two-site ELISA employing anti-histone as the primary antibody and anti-DNA as the secondary antibody according to the manufacturer's instructions (Boehringer Mannheim, TerreHoite, CA, USA). Cells were incubated for different times periods and cells ($1 \times 10^4$ cells $ml^{-1}$) were transferred into sterile 1.5-ml microcentrifuge tubes. Cells were then spun, resuspended in 500 µl of lysis buffer and incubated for 30 min on ice. After centrifugation, the supernatants (cytosol-containing low molecular weight, fragmented DNA) were diluted 1:10 (v:v) with incubation buffer and 100 µl of the solution ($1 \times 10^3$ cell equivalents per ml) pipetted into the wells of a 96-well plate precoated with anti-histone antibody. After incubation and washing, the secondary antibody (anti-DNA) conjugated with horseradish peroxidase was added to the wells. At the end of an additional period of incubation, the wells were treated with chromogen substrate and the intensity of the colour development was assayed with an ELISA plate reader at a 405/490 nm wavelength. Each experiment was performed in triplicate.

### Statistical analysis

The unpaired *t*-test was used to determine the statistical differences. A *P*-value of less than 0.05 was designated to be statistically significant.

### RESULTS

### The levels of gene expression and secretion of cytokines in the cancer cells

Cells were incubated for 48 h and the gene expressions of IL-1β, IL-1ra and ICE were assessed by Northern blot analysis. IL-1β gene was expressed at the same levels in all cancer cells. In contrast, the gene expressions of IL-1ra and ICE in KU-19-19 and KU-19-20 were suppressed compared with those in KU-1 and KU-2 (Figure 1A). Western blot analysis showed that the icIL-1ra (about 20 kDa) was detected at the same levels in all cancer cells. The bands corresponding to immature form pIL-1β (about 33 kDa) were strongly detected in KU-19-19 and KU-19-20, whereas smaller bands were detected in KU-1 and KU-2 (Figure 1B). ELISA showed that the concentrations of both IL-1β and sIL-1ra in the supernatants and IL-1ra/IL-1β ratios were higher in KU-1 and KU-2 than in KU-19-19 and KU-19-20. On the other hand, IL-6, IL-8, G-CSF and GM-CSF were secreted in large quantities in both KU-19-19 and KU-19-20, but not in either KU-1 or KU-2 (Table 1).

### Effect of exogenous IL-1β on the cell growth and cytokine secretion

The cancer cells ($1 \times 10^3$ per well) were treated with 10 ng $ml^{-1}$ IL-1β and incubated for 48 h and then the effect of exogenous IL-1β on

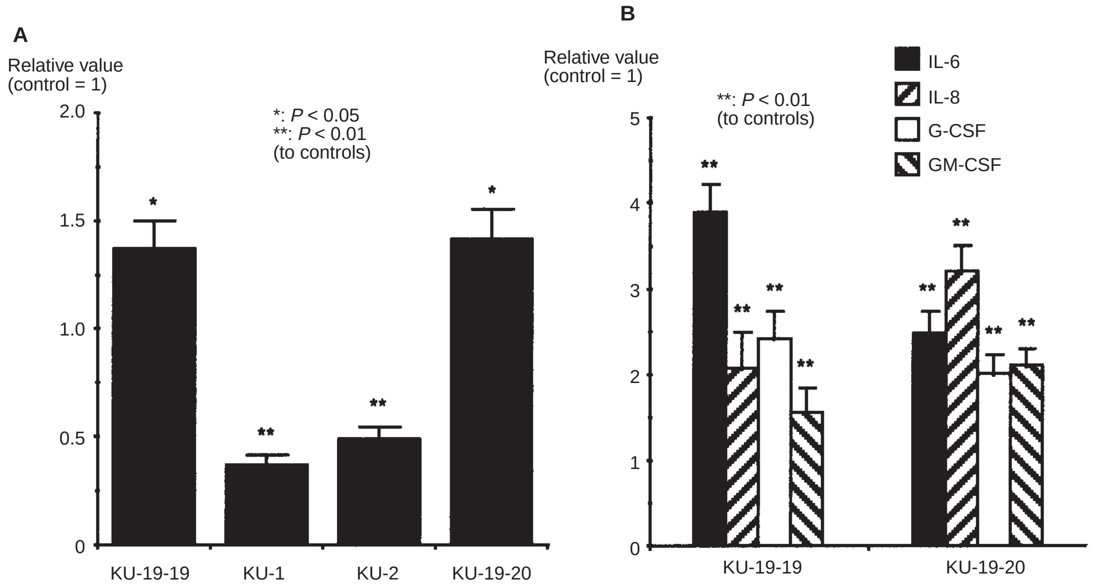

**Figure 2** Effect of exogenous IL-1β on cell survival and cytokine secretion. (**A**) The cancer cells ($1 \times 10^3$ per well) were treated with 10 ng/ml IL-1β and incubated for 48 h and the effect of exogenous IL-1β on the growth of these cancer cells was examined by MTT assay. IL-1β treatment stimulated cell growth in both KU-19-19 and KU-19-20 ($P < 0.05$), while it induced a dramatic growth inhibition in KU-1 and KU-2 ($P < 0.01$). (**B**) KU-19-19 and KU-19-20 cells ($1 \times 10^4$ per well) were treated with 10 ng $ml^{-1}$ IL-1β and incubated for 48 h and the levels of several cytokines in the supernatants were measured by ELISA. The levels of cytokines in the supernatants were elevated significantly more in both KU-19-19 and KU-19-20 than in the non-treated controls ($P < 0.01$). The results are expressed as the relative ratio to the non-treated controls. Bars, s.d. of triplicate samples. The experiments were repeated three times with similar results

© 1999 Cancer Research Campaign

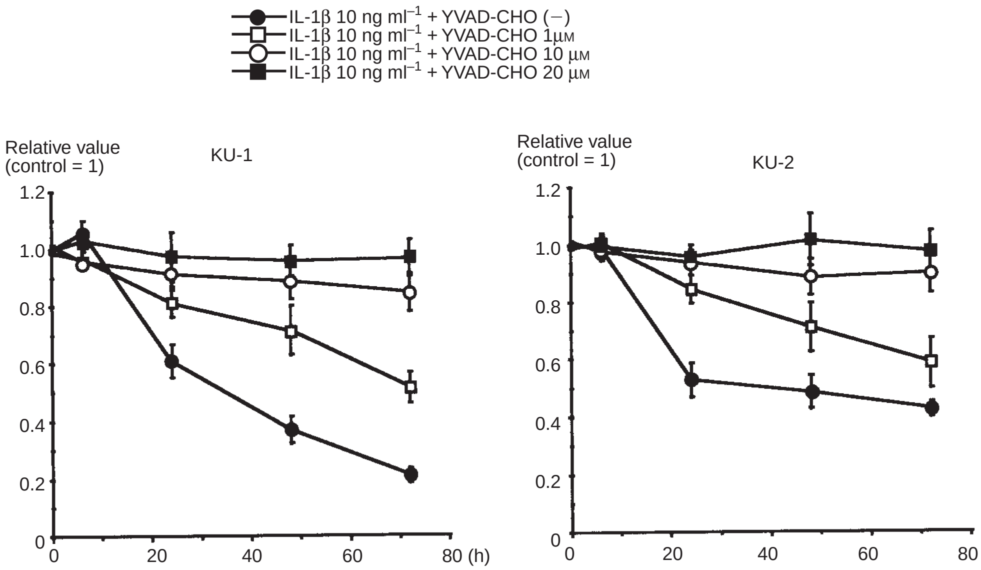

**Figure 3** ICE is required for IL-1β-induced cell death in KU-1 and KU-2. KU-1 and KU-2 cells ($1 \times 10^3$ per well), treated with 10 ng $ml^{-1}$ IL-1β and various concentrations of YVAD-CHO, a reversible ICE inhibitor, were incubated for different time periods and the cell growth was estimated by MTT assay. YVAD-CHO treatment blocked IL-1β-induced growth inhibition dose-dependently in both KU-1 and KU-2. The results are expressed as the relative ratio to the non-treated controls. Bars, s.d. of triplicate samples. The experiments were repeated three times with similar results

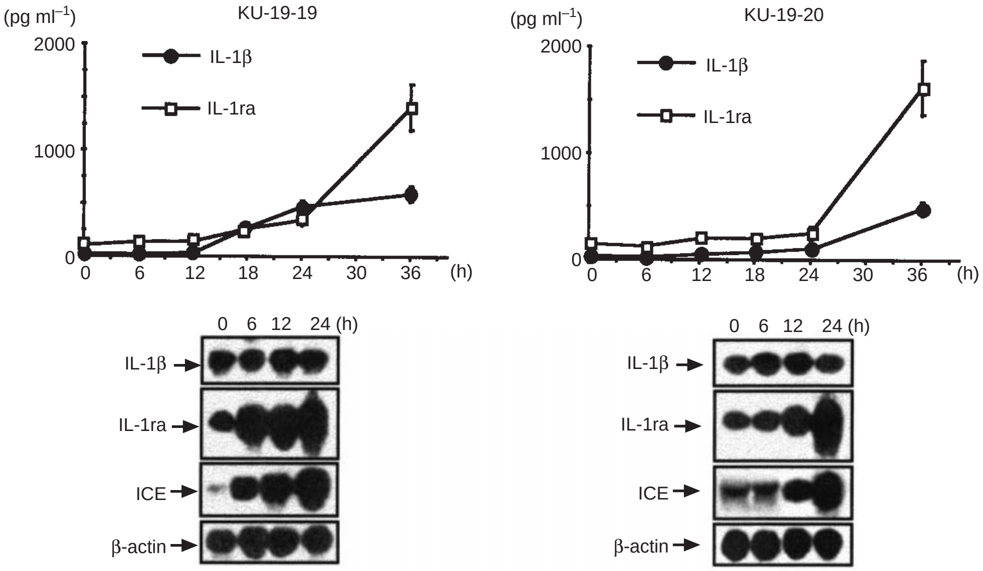

**Figure 4** Effect of AxIL-1ra on ICE gene expression and IL-1β secretion in KU-19-19 and KU-19-20. Cells ($1 \times 10^4$ per well), treated with AxIL-1ra at a concentration of 5 MOI, were incubated for different time periods and the levels of IL-1ra and IL-1β in the supernatants were examined by ELISA. Furthermore, the levels of IL-1β, IL-1ra and ICE gene expression were examined by Northern blot analysis. The IL-1ra gene overexpression was detected 12 h later in KU-19-19 and 18 h later in KU-19-20. The level of IL-1β gene expression did not change in either KU-19-19 or KU-19-20. The ICE gene expression was, however, enhanced according to the IL-1ra gene overexpression. Furthermore, the concentrations of IL-1β as well as IL-1ra in the supernatants dramatically increased after the strong gene expressions of IL-1ra and ICE in both KU-19-19 and KU-19-20. The results of ELISA are expressed as the mean ± s.d. of triplicate samples. Bars, s.d. The experiments were repeated twice with similar results the growth of these cancer cells was examined by MTT assay. As shown in Figure 2A, IL-1β treatment stimulated cell growth in both KU-19-19 and KU-19-20 significantly compared with non-treated controls ($P < 0.05$), while it induced a dramatic growth inhibition in KU-1 and KU-2 ($P < 0.01$). Furthermore, KU-19-19 and KU-19-20 ($1 \times 10^4$ per well) were treated with 10 ng $ml^{-1}$ IL-1β and incubated for 48 h and the levels of several cytokines in the supernatants were measured by ELISA. The levels of cytokines in the supernatants were elevated significantly more in both KU-19-19 and KU-19-20 than in the non-treated controls ($P < 0.01$, Figure 2B).

*© 1999 Cancer Research Campaign* 

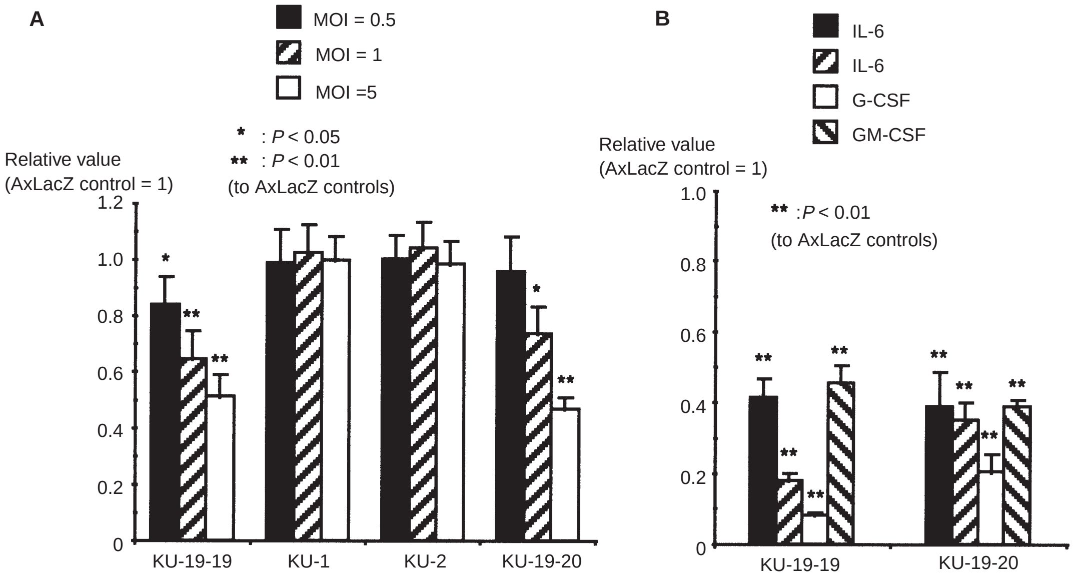

**Figure 5** Effect of AxIL-1ra on the cell growth and cytokine secretion. (**A**) The cancer cells ($1 \times 10^3$ per well), treated with various concentrations of AxIL-1ra or AxLacZ were incubated for 48 h and cell survival was examined by MTT assay. AxIL-1ra-treated cells showed significant growth inhibition on both KU-19-19 and KU-19-20 in dose-dependent fashion compared with AxLacZ-treated control cells. On the other hand, neither KU-1 nor KU-2 treated AxIL-1ra showed significant cell damage compared with AxLacZ-treated control cells. (**B**) KU-19-19 and KU-19-20 cells ($1 \times 10^4$ per well), treated with 5 MOI of AxIL-1ra or AxLacZ, were incubated for 48 h and the levels of several cytokines in the supernatants were measured by ELISA. The relative values of the concentrations of several cytokines in the supernatants in AxIL-1ra-treated cells (MOI = 5) decreased significantly compared with those in the AxLacZ-treated controls in both KU-19-19 and KU-19-20. The results are expressed as the relative ratio to the AxLacZ-treated controls. Bars, s.d. of triplicate samples. The experiments were repeated three times with similar results

### ICE is required for IL-1β-induced growth inhibition in KU-1 and KU-2

We investigated whether or not ICE is required for IL-1β-induced growth inhibition using an ICE inhibitor YVAD-CHO. KU-1 and KU-2 cells ($1 \times 10^3$ per well), treated with 10 ng $ml^{-1}$ IL-1β and various concentrations of YVAD-CHO, were incubated for different time periods and cell survival was estimated by MTT assay. YVAD-CHO treatment blocked IL-1β-induced growth inhibition in a dose-dependent fashion in both KU-1 and KU-2 (Figure 3).

### Effect of AxIL-1ra on ICE gene expression and IL-1β secretion in KU-19-19 and KU-19-20

The results of Figure 1 and Table 1 suggested that the IL-1ra expression might regulate the ICE expression. We therefore investigated whether endogenous sIL-1ra overexpression could enhance the ICE gene expression in KU-19-19 and KU-19-20. Cells, treated with AxIL-1ra at a concentration of 5 multiplicity of infection (MOI), were incubated for different time periods and the gene expressions of IL-1β, IL-1ra and ICE were examined by Northern blot analysis. Furthermore, the levels of IL-1ra and IL-1β secretion in the supernatants were examined by ELISA. The IL-1ra gene overexpression was detected 12 h later in KU-19-19 and 18 h later in KU-19-20. The ICE gene expression was enhanced according to the IL-1ra gene overexpression. Furthermore, the concentrations of IL-1β as well as IL-1ra in the supernatants dramatically increased after the overexpression of IL-1ra and ICE in both KU-19-19 and KU-19-20, whereas the level of IL-1β gene expression did not change in either cell lines (Figure 4). AxLacZ treatment did not change the levels of either the ICE gene expression or IL-1β secretion (data not shown). Although the ICE gene expression was also enhanced in KU-1 and KU-2 treated with AxIL-1ra, the concentration of IL-1β did not dramatically increase (data not shown).

### Effect of AxIL-1ra on the cell growth and the secretion of several cytokines

We investigated whether AxIL-1ra treatment could have some effect on the growth of the cancer cells. The cancer cells ($1 \times 10^3$ per well), treated with various concentrations of AxIL-1ra were incubated for 48 h and the cell growth was examined by MTT assay. As shown in Figure 5A, AxIL-1ra-treated cells showed significant growth inhibition on both KU-19-19 and KU-19-20 in dose-dependent fashion compared with AxLacZ-treated control cells ($P < 0.05$). On the other hand, neither KU-1 nor KU-2 treated with AxIL-1ra showed significant growth inhibition compared with AxLacZ-treated control cells. Furthermore, KU-19-19 and KU-19-20 cells ($1 \times 10^4$ per well), treated with 5 MOI of AxIL-1ra or AxLacZ, were incubated for 48 h and the levels of several cytokines in the supernatants were measured by ELISA. The relative values of their concentrations in the supernatants in the AxIL-1ra-treated cells (MOI = 5) decreased significantly compared with those in the AxLacZ-treated controls in both KU-19-19 and KU-19-20 ($P < 0.01$, Figure 5B). On the other hand, the concentration of IL-1β was more than 100-fold elevated in both cell lines (data not shown).

### Exogenous IL-1ra can not induce either ICE gene expression or growth inhibition, while it can block the secretion of cytokines in KU-19-19 and KU-19-20

Considering the possibility that the surplus amount of extracellular sIL-1ra derived from AxIL-1ra might promote ICE gene expres-

© 1999 Cancer Research Campaign

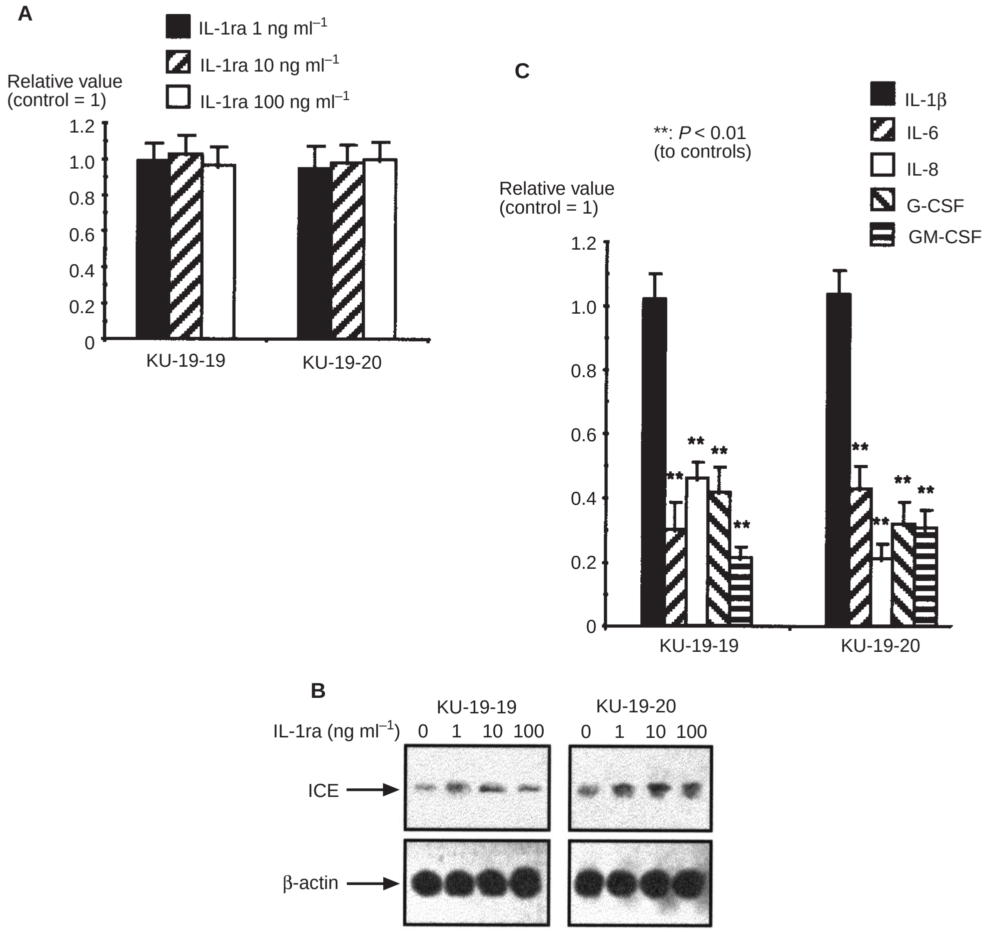

**Figure 6** Effect of exogenous IL-1ra on the cell growth, ICE gene expression and cytokine secretion in KU-19-19 and KU-19-20. (**A**) Cells ($1 \times 10^3$ per well), treated with various concentrations of IL-1ra were incubated for 48 h and cell survival was estimated by MTT assay. IL-1ra at the concentrations of 100 ng $ml^{-1}$ or less had no effect of the cell growth in either KU-19-19 or KU-19-20. The results are expressed as the relative ratio to the non-treated controls. Bars, s.d. of triplicate samples. (**B**) The level of ICE gene expression by exogenous IL-1ra treatment. Northern blot analysis showed that IL-1ra treatment (100 ng $ml^{-1}$) or less could not enhance the ICE gene expression in either KU-19-19 or KU-19-20. (**C**) Cells ($1 \times 10^4$ per well), treated with various concentrations of IL-1ra were incubated for 48 h and the levels of several cytokines in the supernatants were measured by ELISA. In both KU-19-19 and KU-19-20, IL-1ra treatment (100 ng $ml^{-1}$) significantly inhibited the secretion of several cytokines but IL-1β compared with non-treatment. The results are expressed as the relative ratio to the non-treated controls. Bars, s.d. of triplicate samples. The experiments were repeated twice with similar results sion and inhibit cell growth, we investigated whether exogenous IL-1ra could regulate ICE gene expression and cell growth in KU-19-19 and KU-19-20. Cells ($1 \times 10^3$ per well), treated with various concentrations of IL-1ra, were incubated for 48 h and cell growth was examined by MTT assay. Furthermore, the level of ICE gene expression was examined by Northern blot analysis. As shown in Figure 6A, IL-1ra at the concentrations of 100 ng $ml^{-1}$ or less had no effect on the cell growth of either KU-19-19 or KU-19-20. In addition, IL-1ra treatment was not able to enhance the ICE gene expression in either KU-19-19 or KU-19-20 (Figure 6B). On the other hand, ELISA study showed IL-1ra treatment (100 ng $ml^{-1}$) inhibited the secretion of several cytokines significantly in both KU-19-19 and KU-19-20 compared with non-treatment 48 h later ($P < 0.01$), while it had little effect on the concentration of IL-1β (Figure 6C). These results suggested that AxIL-1ra-induced ICE expression, IL-1β secretion and growth inhibition did not result from extracellular sIL-1ra and that the decrease of the concentrations of several cytokines but IL-1β had little effect on the cell growth in either KU-19-19 or KU-19-20 during the observation period.

### AxIL-1ra induces apoptotic cell death on both KU-19-19 and KU-19-20

We furthermore investigated whether AxIL-1ra-induced growth inhibition could result from apoptotic cell death in KU-19-19 and KU-19-20 by fragmented DNA ELISA. In KU-19-19, absorbance was elevated significantly more in the AxIL-1ra (MOI = 5)-treated cells than in the AxLacZ (MOI = 5)-treated cells 24 h later and it was dramatically elevated 48 h later ($P < 0.01$), indicating the presence of the low molecular weight fragmented DNA in the cytosolic compartment. In KU-19-20, it was also significantly elevated 48 h later ($P < 0.01$, Figure 7).

### Both IL-1β and ICE are required for AxIL-1ra-induced cell death in KU-19-19 and KU-19-20

We investigated whether both IL-1β and ICE are required to induce growth inhibition on KU-19-19 and KU-19-20. AxIL-1ra (MOI = 5)-infected cells ($1 \times 10^3$ per well) were treated with either IL-1ra (100 ng $ml^{-1}$), anti-IL-1β antibody (10 μg $ml^{-1}$) or YVAD-CHO

© 1999 Cancer Research Campaign 

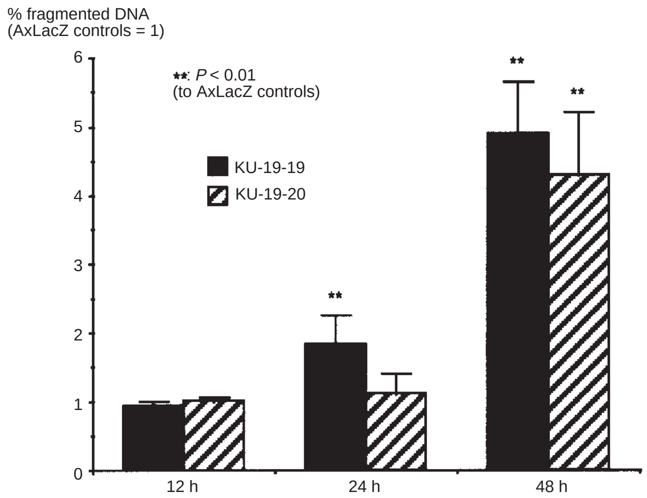

**Figure 7** Measurement of AxIL-1ra-induced apoptosis in KU-19-19 and KU-19-20 by fragmented DNA ELISA. Cells ($1 \times 10^4$ cells $ml^{-1}$), treated with AxIL-1ra (MOI = 5) or AxLacZ (MOI = 5) for different time periods, were transferred into sterile 1.5-ml microcentrifuge tubes. After cells were then lysed and centrifuged, 100 μl of tenfold diluted supernatants ($1 \times 10^3$ cell equivalents $ml^{-1}$) was pipetted into the 96-well plate and the level of the fragmented DNA was measured by specific two-site ELISA. In KU-19-19, absorbance was elevated significantly more in the AxIL-1ra (MOI = 5)-treated cells than in the AxLacZ (MOI = 5)-treated cells 24 h later and it was dramatically elevated 48 h later ($P < 0.01$). In KU-19-20, it was also significantly elevated 48 h later ($P < 0.01$). The results are expressed as the relative ratio to the AxLacZ-treated controls. Bars, s.d. of triplicate samples. The experiments were repeated three times with similar results

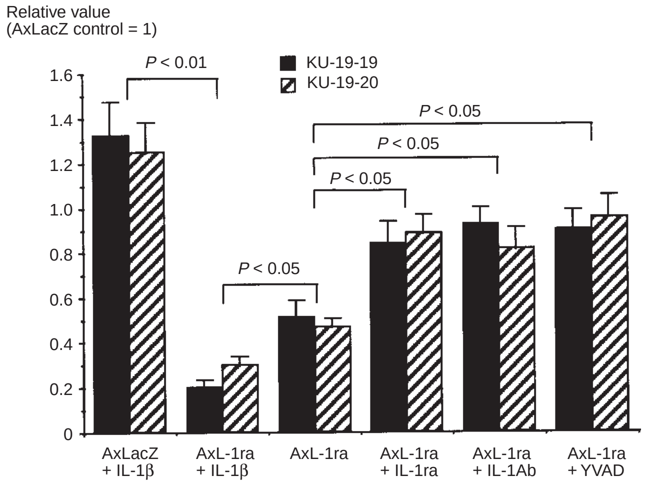

**Figure 8** Both IL-1β and ICE function are required for AxIL-1ra-induced cell death in KU-19-19 and KU-19-20. AxIL-1ra (MOI = 5)-infected cells ($1 \times 10^3$ per well) were treated with either IL-1ra (100 ng $ml^{-1}$), anti-IL-1β antibody (IL-1β Ab; 10 μg $ml^{-1}$) or YVAD-CHO (20 μM), and then were incubated for 48 h, and cell survival was estimated by MTT assay. These inhibitors significantly blocked AxIL-1ra-induced cell death in both KU-19-19 and KU-19-20 ($P < 0.05$). IL-1β (10 ng $ml^{-1}$) could enhance AxIL-1ra-induced growth inhibition in both KU-19-19 and KU-19-20 ($P < 0.05$), while it induced growth promotion under AxLacZ treatment. The results are expressed as the relative ratio to the AxLacZ-treated controls. Bars, s.d. of triplicate samples. The experiments were repeated twice with similar results

(20 μM), and then were incubated for 48 h, and the cell growth was estimated by MTT assay. Both IL-1ra and anti-IL-1β antibody were used as the inhibitors of IL-1β in the supernatants and YVAD-CHO as an inhibitor of ICE. They significantly blocked AxIL-1ra-induced growth inhibition in both KU-19-19 and KU-19-20 ($P < 0.05$). Furthermore, IL-1β (10 ng $ml^{-1}$) could further enhance AxIL-1ra-induced growth inhibition in both KU-19-19 and KU-19-20 ($P < 0.05$), while it induced growth promotion under AxLacZ treatment (Figure 8). YVAD-CHO (20 μM or less) only did not effect the cell growth in either KU-19-19 or KU-19-20 (data not shown).

### DISCUSSION

Many authors have reported that cell resistance to the antiproliferative effect of IL-1 appears to constitutively produce IL-1, which may be associated with cell proliferation including autocrine/paracrine growth and thus promote the production of other cytokines (Onozaki et al, 1985; Ito et al, 1993; Araki et al, 1994; Fratelli et al, 1995; Furukawa et al, 1995). In the present study, we first investigated whether or not IL-1β-producing cells are really resistant to IL-1β. We demonstrated that IL-1β-sensitive cancer cell lines, KU-1 and KU-2, produce IL-1β. Surprisingly, the IL-1β concentrations in the supernatants were rather lower in IL-1β-sensitive cancer cell lines. This finding indicates that endogenously IL-1β producing cells are thus not necessarily resistant to IL-1β.

We next investigated whether IL-1β-sensitivity is dependent on the intracellular ICE expression. As expected, in IL-1β-resistant cancer cell lines, KU-19-19 and KU-19-20, ICE gene expression was found to be suppressed compared with KU-1 and KU-2 and an ICE inhibitor YVAD-CHO inhibited IL-1β-induced cell death in KU-1 and KU-2. These results suggest that ICE inhibition could cause the resistance to IL-1β.

Furthermore, we showed that both IL-1ra and IL-1ra and IL-1β antibody, which inhibit IL-1β-IL-1 receptor interaction, blocked AxIL-1ra-induced cell death in KU-19-19 and KU-19-20. This result strongly suggests that IL-1β is essential for AxIL-1ra-induced cell death. We showed pIL-1β to be accumulated in large quantities in both KU-19-19 and KU-19-20, but not in either KU-1 or KU-2 by Western blot analysis and that a great deal of IL-1β was secreted all at once by AxIL-1ra treatment in both KU-19-19 and KU-19-20. Previous works have also demonstrated that mIL-1β is secreted during apoptosis mediated via a variety of stimuli (Hogquist et al, 1991; Miura et al, 1993, 1995; Zychlinsky et al, 1994). Friedlander et al (1996) reported that the cleavage and secretion of endogenous IL-1β plays an important role in ICE-mediated cell death.

Many authors have recently reported that endogenous IL-1/IL-1ra balance regulates the homeostasis of cell proliferation and cell death (Corradi et al, 1995; Furukawa et al, 1995; Goletti et al, 1996; Oelmann et al, 1997). In various types of cells, the production and secretion of IL-1 and IL-1ra is simultaneous. In fact, all the cancer cell lines used in this study express IL-1ra as well as IL-1β. While IL-1ra is secreted through the classical exocytotic pathway, IL-1β lacks a secretory signal peptide and its secretion thus depends on the ICE activity. It is thought to be quite a natural homeostatic mechanism to block IL-1β secretion in case IL-1ra expression is suppressed compared with IL-1β expression. While previous authors referred to the extracellular IL-1/IL-1ra balance, we have demonstrated that intracellular IL-1β/IL-1ra gene expression balance plays a crucial role in the cell proliferation and death. In this study, we demonstrated that the surplus amount of exogenous IL-1ra could promote neither ICE gene expression nor IL-1β

 © 1999 Cancer Research Campaign secretion in KU-19-19 and KU-19-20, while endogenous IL-1ra overexpression promotes both ICE gene expression and IL-1β secretion. Furthermore, Figure 4 shows that AxIL-1ra-induced ICE gene overexpression occurred earlier than increase of the IL-1ra concentration in the supernatants in both KU-19-19 and KU-19-20. We demonstrated that icIL-1ra protein levels were not different in four cancer cell lines as shown in Figure 1B and furthermore confirmed that AxIL-1ra did not increase icIL-1ra protein levels in either KU-19-19 or KU-19-20 (data not shown). These results strongly support the idea that the regulation of ICE gene expression is likely due to an intracellular function of sIL-1ra rather than to either icIL-1ra function or IL-1 receptor-mediated extracellular event. As far as we investigated, this is a first report on the intracellular function of sIL-1ra. However, it remains unclear whether sIL-1ra can directly regulate the level of ICE gene expression before it is secreted or some other co-factors play a crucial role in ICE regulation at the transcription level of sIL-1ra, and the further study will be needed to clarify this mechanism.

It is well known that a 10- to 100-fold molar excess of IL-1ra is often required to inhibit IL-1 activity (Arend et al, 1990). In the present study, we found that IL-1ra/IL-1β ratios in the supernatants were less than 10 in KU-19-19 and KU-19-20, while those were more than 10 in KU-1 and KU-2. This result suggests that in KU-19-19 and KU-19-20, IL-1β can overcome IL-1ra at the non-treated condition. IL-1β is well known as one of the key cytokine that can promote the production of various cytokines including IL-6, IL-8, G-CSF and GM-CSF (Dinarello, 1996). Some of these cytokines might help cell proliferation by an autocrine and/or paracrine loop (Tachibana et al, 1995) and in fact, high-dose IL-1β treatment could promote the production of various cytokines and cell proliferation in both KU-19-19 and KU-19-20. In contrast, IL-1β treatment blocked cell proliferation in KU-1 and KU-2, and furthermore it also blocked cell proliferation in AxIL-1ra-treated KU-19-19 and KU-19-20. Taken together, it is suggested that IL-1β is associated with both cell proliferation and cell death and that its function is dependent on the ICE expression. In KU-1 and KU-2, in which ICE is activated since the endogenous IL-1ra/IL-1β expression ratio is high, IL-1β functions as an apoptosis inducer. In contrast, in KU-19-19 and KU-19-20 in which ICE is inhibited since the endogenous IL-1ra/IL-1β expression ratio is low, IL-1β can not function as an apoptosis inducer but can promote various types of cytokine production which thus induce cell proliferation by an autocrine and/or paracrine loop. Several reports have referred to this dual function of IL-1β. Fratelli et al (1995) reported that IL-1β promotes proliferation during the exponential phase, while it induces apoptosis during the plateau phase of cell growth in thymoma cells and that a signal exists which converts the cellular response to IL-1β from proliferation to death. Furthermore, Friedlander et al (1996) reported that the ICE down-regulates IL-1β-IL-1 receptor-binding activity, which causes the resistance to IL-1β. In the preliminary study by receptor-binding assay, however, we did not find this phenomenon in either KU-19-19 or KU-19-20 (data not shown).

In conclusion, we demonstrated that the resistance to IL-1β does not necessarily result from the constitutive IL-1β production but depends on the level of ICE gene expression, which is regulated by the level of endogenous sIL-1ra production. Since IL-1β functions as an inducer of various cytokines which can promote cancer cell proliferation as indicated in this study, these findings, therefore, provide key insights into the mechanism underlying the viability of cancer cells.

### ACKNOWLEDGEMENTS

This work was supported in part by grants-in-aid Nos 07407046 and 10146673 for Scientific Research from the Ministry of Education, Science and Sport Culture, Japan.

### REFERENCES

Alnemri ES, Livingston DJ, Nicholson DW, Salvesen G, Thornberry NA, Wong WW and Yuan J (1996) Human ICE/CD-3 protease nomenclature [letter]. *Cell* **87**: 171

Araki M, Yano T, Hayashi H, Takii T, Suzuki K and Onozaki K (1994) Resistance to the anti-proliferative effect of IL-1 on human melanoma cell line is associated with endogenous production of IL-1 and IL-6. *Int J Cancer* **56**: 275–280

Arend WP, Welgus HG, Thompson RC and Eisenberg SP (1990) Biological properties of recombinant human monocyte-derived interleukin-1 receptor antagonist. *J Clin Invest* **85**: 1694

Black RA, Kronheim SR and Sleath PR (1989) Activation of interleukin-1 beta by a co-induced protease. *FEBS Lett* **247**: 386–390

Cerretti DP, Kozlosky CJ, Mosley B, Nelson N, Van NK and Greenstreet TA (1992) Molecular cloning of the interleukin-1 beta converting enzyme. *Science* **256**: 97–100

Corradi A, Franzi AT and Rubartelli A (1995) Synthesis and secretion of interleukin-1α and interleukin-1 receptor antagonist during differentiation of cultured keratinocytes. *Exp Cell Res* **217**: 355–362

Dinarello CA (1996) Biologic basis for interleukin-1 in disease. *Blood* **87**: 2095–2147

Dinarello CA and Thompson RC (1991) Blocking IL-1:IL-1RA in vivo and in vitro. *Immunol Today* **12**: 404–410

Dinarello CA and Wolff SM (1993) The role of interleukin-1 disease. *N Engl J Med* **328**: 106–113

Enari M, Talanian RV, Wong WW and Nagata S (1996) Sequential activation of ICE-like and CPP-like proteases during Fas-mediated apoptosis. *Nature* **380**: 723–726

Fratelli M, Gagliardini V, Galli G, Gnocchi P, Ghiara P and Ghezzi P (1995) Autocrine interleukin-1β regulates both proliferation and apoptosis in EL4-6.1 thymoma cells. *Blood* **85**: 3532–3537

Freedman AS, Freeman G, Whitman J, Sejil J, Dley J and Nadler LM (1988) Pre-exposure of human B cells to recombinant IL-1 enhances subsequent proliferation. *J Immunol* **141**: 3398–3404

Friedlander RM, Gagliardini V, Rotello RJ and Yuan J (1996) Functional role of interleukin 1β-converting enzyme-mediated apoptosis. *J Exp Med* **184**: 717–724

Furukawa Y, Kikuchi J, Terui Y, Kitagawa S, Ohta M, Miura Y and Saito M (1995) Preferential production of interleukin-1β over interleukin-1 receptor antagonist contributes to proliferation and suppression of apoptosis in leukemia cells. *Jpn J Cancer Res* **86**: 208–216

Gaffney EV and Tsai SC (1986) Lymphocyte-activating and growth-inhibitory activities for several sources native and recombinant interleukin 1. *Cancer Res* **46**: 3834–3837

Gery I, Gershon RK and Waksman BH (1972) Potentiation of the T-lymphocyte responce to mitogens. I. The responding cell. *J Exp Med* **136**: 128–142

Goletti D, Kinter AL, Hardy EC, Poli G and Fauci AS (1996) Modulation of endogenous IL-1β and IL-1 receptor antagonist results in opposing effects on HIV expression in chronically infected monocytic cells. *J Immunol* **156**: 3501–3508

Grassi J, Roberge CJ, Frobert Y, Pradelles P and Poubelle PE (1991) Determination of IL-1α, IL-1β, and IL-2 in biological media using specific enzyme immunometric assays. *Immunol Rev* **119**: 125–145

Hazuda DJ, Lee JC and Young PR (1988) The kinetics of interleukin 1 secretion from activated monocytes. *J Biol Chem* **263**: 8473–8479

Hogquist AH, Nett MA, Unanue ER and Chaplin DD (1991) Interleukin 1 is processed and released during apoptosis. *Proc Natl Acad Sci USA* **88**: 8485–8489

Ito R, Kitadai Y, Kyo E, Yokozaki H, Yasui W, Yamashita U, Nikai H and Tahara E (1993) Interleukin-1α acts as autocrine growth stimulator for human gastric carcinoma cells. *Cancer Res* **53**: 4102–4106

Kanegae Y, Makimura M and Saito I (1994) A simple and efficient method for purification of infectious recombinant adenovirus. *Jpn J Med Sci Biol* **47**: 157–166

Kanegae Y, Lee G, Sato Y, Tanaka M, Nakai M, Sakaki T, Sugano S and Saito I (1995) Efficient gene activation in mammalian cells by using recombinant

© 1999 Cancer Research Campaign 

adenovirus expressing site-specific Cre recombinase. *Nucleic Acids Res* **23**: 3816–3821
Katsuoka Y, Baba S, Hata M and Tazaki H (1976) Transplantation of human renal cell carcinoma to the nude mouse: as an intermediate of in vivo and in vitro studies. *J Urol* **115**: 373–376
Kostura MJ, Tocci MJ, Limjuco G, Chin J, Cameron P and Hillman AG (1989) Identification of a monocyte specific pre-interleukin-1 beta convertase. *Proc Natl Acad Sci USA* **86**: 5227–5231
Lachman LB, Brown DC and Dinarello CA (1987) Growth-promoting effect of recombinant interleukin 1 and tumor necrosis factor for a human astrocytoma cell line. *J Immunol* **138**: 2913–2916
Lonnenmann G, Endras S, Van der Meer JW, Cannon JG, Koch KM and Dinarello CA (1989) Differences in the synthesis and kinetics of release of interleukin-1. *Eur J Immunol* **19**: 1531–1536
Maier JAM, Voulalas P, Roeder D and Maciag T (1990) Extension of the life-span of human endothelial cells by an interleukin-1α antisense oligomer. *Science* **249**: 1570–1574
Miura M, Zhu H, Rottelo R, Hartweig E and Yuan J (1993) Induction of apoptosis in fibroblasts by IL-1β-converting enzyme, a mammalian homolog of the *C. elegans* cell death gene ced-3. *Cell* **75**: 641–652
Miura M, Friedlander RM and Yuan J (1995) Tumor necrosis factor-induced apoptosis is mediated by a CrmA-sensitive cell death pathway. *Proc Natl Acad Sci USA* **92**: 8318–8322
Miyake S, Makimura M, Kanegae Y, Harada S, Sato Y, Takamori K, Tokuda C and Saito I (1996) Efficient generation of recombinant adenoviruses using adenovirus DNA-terminal protein complex and a cosmid bearing the full-length virus genome. *Proc Natl Acad Sci USA* **93**: 1320–1324
Oelmann E, Kraemer A, Serve H, Reufi B, Oberberg D, Patt S, Herbst H, Stein H, Thiel E and Berdel WE (1997) Autocrine interleukin-1 receptor antagonist can support malignant growth of glioblastoma by blocking growth-inhibiting autocrine loop of interleukin-1. *Int J Cancer* **71**: 1066–1076
Onozaki K, Matsushima K, Aggarwal BB and Oppenheim JJ (1985) Human interleukin 1 is a cytocidal factor for several tumor cell lines. *J Immunol* **135**: 3962–3968
Oppenheim JJ, Kovacs EJ, Matsushima K and Durum SK (1986) There is more than one interleukin-1. *Immunol Today* **7**: 45–56
Schmidt JA, Mizel SB, Cohen D and Green I (1982) Interleukin-1, a potent regulator of fibroblast proliferation. *J Immunol* **128**: 2177–2182
Smith DJ, McGuire MJ, Tocci MJ and Thiele DL (1997) IL-1β converting enzyme (ICE) does not play a requisite role in apoptosis induced in T lymphoblasts by Fas-dependent or Fas-independent CTL effector mechanisms. *J Immunol* **158**: 163–170
Tachibana M (1982) Studies on cellular adhesiveness in five different culture cell lines derived from carcinoma of the urinary bladder. *Keio J Med* **31**: 127–148
Tachibana M, Miyakawa A, Tazaki H, Nakamura K, Kubo A, Hata J, Nishi T and Amano Y (1995) Autocrine growth of transitional cell carcinoma of the bladder induced by granulocyte-colony stimulating factor. *Cancer Res* **55**: 3438–3443
Tachibana M, Miyakawa A, Nakashima J, Murai M, Nakamura K, Kubo A and Hata J-I (1997*a*) Constitutive production of multiple cytokines and a human chorionic gonadotrophin beta-subunit by a human bladder cancer cell line (KU-19-19): possible demonstration of totipotential differentiation. *Br J Cancer* **76**: 163–174
Tachibana M, Miyakawa A, Uchida A, Murai M, Eguchi K, Nakamura K, Kubo A and Hata J-I (1997*b*) Granulocyte colony-stimulating factor receptor expression on human transitional cell carcinoma of the bladder. *Br J Cancer* **75**: 1489–1496
Tatsuda T, Cheng J and Mountz JD (1996) Intracellular IL-1β is an inhibitor of Fas-mediated apoptosis. *J Immunol* **157**: 3949–3957
Zychlinsky A, Fitting C, Cavaillon JM and Sansonetti PJ (1994) Interleukin 1 is released by murine macrophages during apoptosis induced by *Shigella flexneri*. *J Clin Invest* **94**: 1328–1332

© 1999 Cancer Research Campaign
